# Single-cell transcriptomic analysis of retinal immune regulation and blood-retinal barrier function during experimental autoimmune uveitis

**DOI:** 10.1038/s41598-024-68401-y

**Published:** 2024-08-28

**Authors:** Joel Quinn, Ahmed Salman, Christopher Paluch, Matthew Jackson-Wood, Michelle E. McClements, Jian Luo, Simon J. Davis, Richard J. Cornall, Robert E. MacLaren, Calliope A. Dendrou, Kanmin Xue

**Affiliations:** 1 Nuffield Laboratory of Ophthalmology, Nuffield Department of Clinical Neurosciences, University of Oxford, Oxford, UK; 2 MiroBio Ltd, Winchester House, Heatley Rd, Oxford, UK; 3 Radcliffe Department of Medicine, John Radcliffe Hospital, University of Oxford, Oxford, UK; 4 Medical Research Council Human Immunology Unit, John Radcliffe Hospital, University of Oxford, Oxford, UK; 5 Respiratory Medicine Unit, Nuffield Department of Medicine, Experimental Medicine, University of Oxford, Oxford, UK; 6 Nuffield Department of Medicine, Henry Wellcome Building for Molecular Physiology, University of Oxford, Oxford, UK; 7 Henry Wellcome Building for Molecular Physiology, CAMS Oxford Institute, University of Oxford, Oxford, UK; 8 Oxford Eye Hospital, Oxford University Hospitals NHS Foundation Trust, Oxford, UK; 9 Nuffield Department of Medicine, Wellcome Centre for Human Genetics, University of Oxford, Oxford, UK; 10 Nuffield Department of Orthopaedics, Rheumatology and Musculoskeletal Sciences, Kennedy Institute of Rheumatology, University of Oxford, Oxford, UK; 11 Nuffield Department of Clinical Neurosciences, John Radcliffe Hospital, University of Oxford, Oxford, UK

**Keywords:** Neuroimmunology, Autoimmunity, Retina

## Abstract

Uveitis is characterised by breakdown of the blood-retinal barrier (BRB), allowing infiltration of immune cells that mediate intraocular inflammation, which can lead to irreversible damage of the neuroretina and the loss of sight. Treatment of uveitis relies heavily on corticosteroids and systemic immunosuppression due to limited understanding of disease pathogenesis. We performed single-cell RNA-sequencing of retinas, as well as bulk RNA-sequencing of retinal pigment epithelial (RPE) cells from mice with experimental autoimmune uveitis (EAU) versus healthy control. This revealed that the Th1/Th17-driven disease induced strong gene expression changes in response to inflammation in rods, cones, Müller glia and RPE. In particular, Müller glia and RPE cells were found to upregulate expression of chemokines, complement factors, leukocyte adhesion molecules and MHC class II, thus highlighting their contributions to immune cell recruitment and antigen presentation at the inner and outer BRB, respectively. Additionally, ligand-receptor interaction analysis with CellPhoneDB revealed key interactions between Müller glia and T cell / natural killer cell subsets via chemokines, galectin-9 to P4HB/TIM-3, PD-L1 to PD-1, and nectin-2/3 to TIGIT signalling axes. Our findings elucidate mechanisms contributing to breakdown of retinal immune privilege during uveitis and identify novel targets for therapeutic interventions.

## Introduction

Uveitis is a major cause of blindness, contributing to 10–15% of severe visual impairment, particularly in the working age^1,2^. It encompasses a group of disease entities that present with inflammation of the iris, ciliary body, vitreous, retina or choroid, which is associated with breakdown of the blood-retinal barrier (BRB) and infiltration of immune cells. While some cases of uveitis are linked to infections (such as *Mycobacterium tuberculosis*), many appear to be autoimmune or autoinflammatory in nature and associated with systemic diseases such as sarcoidosis, HLA-B27-associated spondyloarthropathies, Behçet’s disease, and multiple sclerosis. Current treatment options for non-infectious and posterior uveitis (inflammation centred on the retina and choroid) rely heavily on systemic immunosuppression, which is hampered by variable efficacy and severe adverse effects^3^. This is in part due to a poor understanding of disease pathogenesis.

Retinal glial cells (mainly Müller glia and some astrocytes) and retinal pigment epithelial (RPE) cells are important constituents of the inner and outer BRB, respectively. These physical barriers tightly regulate the movement of immune cells and molecules in and out of the neuroretina, thus reducing the risk of inflammatory damage of the non-regenerating neurons^4,5^. Previous work has suggested that Müller glia and RPE have direct suppressive effects on lymphocyte activation^6–8^, although this biological aspect of the BRB remains poorly understood. Characterization of changes in gene expression in the BRB and retinal cells between the physiological state and ocular inflammation could help reveal key immune interactions and identify therapeutic targets for immune interventions.

The advent of single-cell RNA-sequencing (scRNA-seq) as a tool for studying gene expression in individual cells has greatly improved our understanding of tissue heterogeneity and enabled in-depth characterization of novel cellular subpopulations. Moreover, differential gene expression analysis of specific cell types in response to disease or stimulus is greatly improved by scRNA-seq, as contaminating populations can be identified and filtered out. This facilitates discovery of key genes and cell types that are important to tissue function or disease pathogenesis. Here, we use a combination of scRNA-seq of the retina and bulk RNA-sequencing of the RPE to study changes in the local immune environment of the murine retina in the most faithful mouse model of human endogenous uveitis. In this model, immunisation against a retinal antigen, interphotoreceptor retinoid-binding protein (IRBP), leads to experimental autoimmune uveitis (EAU) which typically onsets between 10–14 days and peaks between 17–22 days in C57BL/6 mice^9–12^. Through differential expression and ligand-receptor interaction analyses, we found that both Müller glia and RPE undergo wide-ranging transcriptional changes and upregulate genes that contribute to immune cell recruitment from the circulation. In addition, both cell types may be capable of antigen-presentation through upregulation of MHC Class II genes which we also confirmed at the protein level. This supports a hypothesis where, during inflammation, BRB cells shift from an anti-inflammatory to a complex state of anti- and pro-inflammatory gene expression, that not only permits but actively promotes leukocyte entry into the retina. Thus, targeting these acquired BRB-leukocyte interactions may represent an attractive local treatment approach for posterior uveitis, and may be applicable to other neuroinflammatory diseases of the central nervous system (CNS) where the similar blood–brain barrier exists.

## Results

### Single-cell transcriptomic analysis of EAU retinas

**Figure 1:**
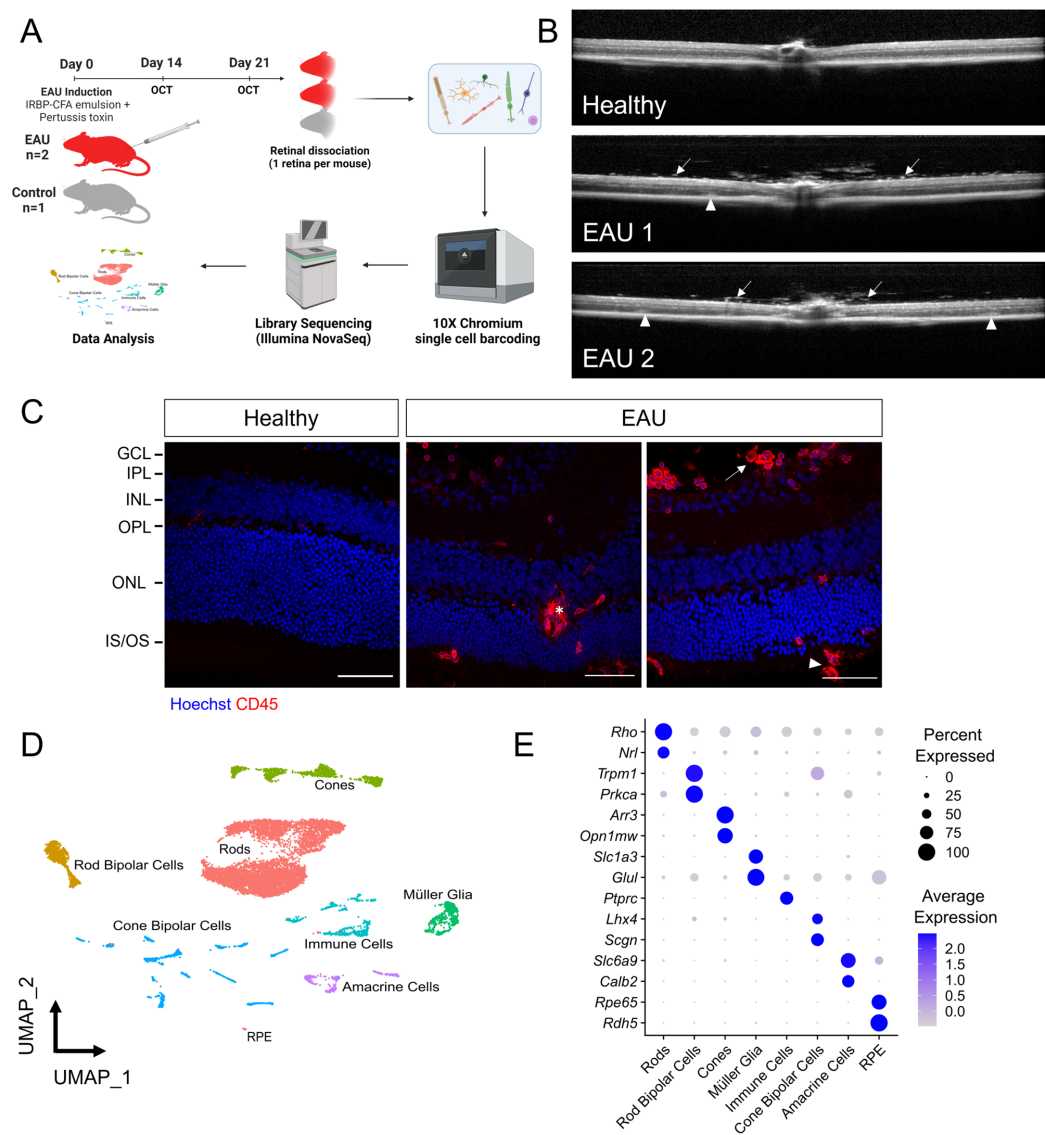
Single-cell transcriptomic analysis of experimental autoimmune uveitis (EAU) versus healthy retinas in mice. (**A**) Study outline. 1 retina per mouse from 2 mice with grade 2 EAU and 1 control littermate mouse (all females) were dissociated 21 days post-immunisation. (**B**) In vivo optical coherence tomography (OCT) of the mouse retinas immediately prior to tissue harvesting for scRNA-seq analysis. Arrows indicate vitreous immune cells; arrowheads indicate subretinal immune infiltrates associated with structural disruption of retinal layers. (**C**) Immunostaining of healthy and EAU retinas for CD45 (in red), showing infiltrating immune cells on the inner retinal surface and within the outer retina in EAU. Asterisk (*) indicates an aggregate of immune cells, likely within an inflamed deep capillary vessel, in the outer plexiform layer (OPL). Arrow indicates immune cells in the vitreous. Arrowhead indicates subretinal immune cells. GCL, ganglion cell layer; IPL, inner plexiform layer; INL, inner nuclear layer; ONL, outer nuclear layer; IS/OS, inner segment-outer segment junction of photoreceptors. (**D**) Integrated UMAP of 11,516 cells from 1 healthy and 2 EAU retinas with annotated cell types. (**E**) Dot plot of marker genes used to identify each cell type.

To explore the transcriptomes of inflamed versus healthy retinas at single cell resolution, 6–10 week-old female C57BL6/J mice were immunised against the retinal antigen peptide IRBP and imaged for the presence of uveitis at day 14 and day 21 post-immunisation (Fig. 1A). At day 21, one healthy retina from an untreated mouse and two retinas with grade 2 EAU^13^(Fig. 1B,C) from two immunised mice were harvested and dissociated into single cells. Droplet-based scRNA-seq (10X Genomics Chromium system) captured a total of 11,516 cells following quality control (Fig. 1D, Supplementary Table S1). Clusters corresponding to retinal cell types were identified using marker genes, including rod photoreceptors (*Rho, Nrl, Nr2e3; n* = *6376 cells*), cone photoreceptors (*Arr3, Opn1mw;* n = 1189 cells), Müller glia (*Slc1a3, Rlbp1, Glul; n* = *622 cells*), rod bipolar cells (*Prkca, Sebox, Trpm1; n* = *919 cells*), cone bipolar cells (*Lhx4, Vsx2, Scgn; n* = *1309 cells*), amacrine cells (*Calb2, Slc6a9;* n = 452 cells), a small cluster of retinal pigment epithelial (RPE) cells (*Rpe65, Rdh5, Rlbp1; n* = *35 cells*), as well as a cluster of immune cells (*Ptprc; n* = *614*) (Fig. 1E, Supplementary Table S2). Annotated retinal cell types were found in similar proportions in the two EAU samples, while non-microglial immune cells came almost exclusively from EAU retinae, as expected (Supplementary Fig. S1, Supplementary Table S3).

**Figure 2:**
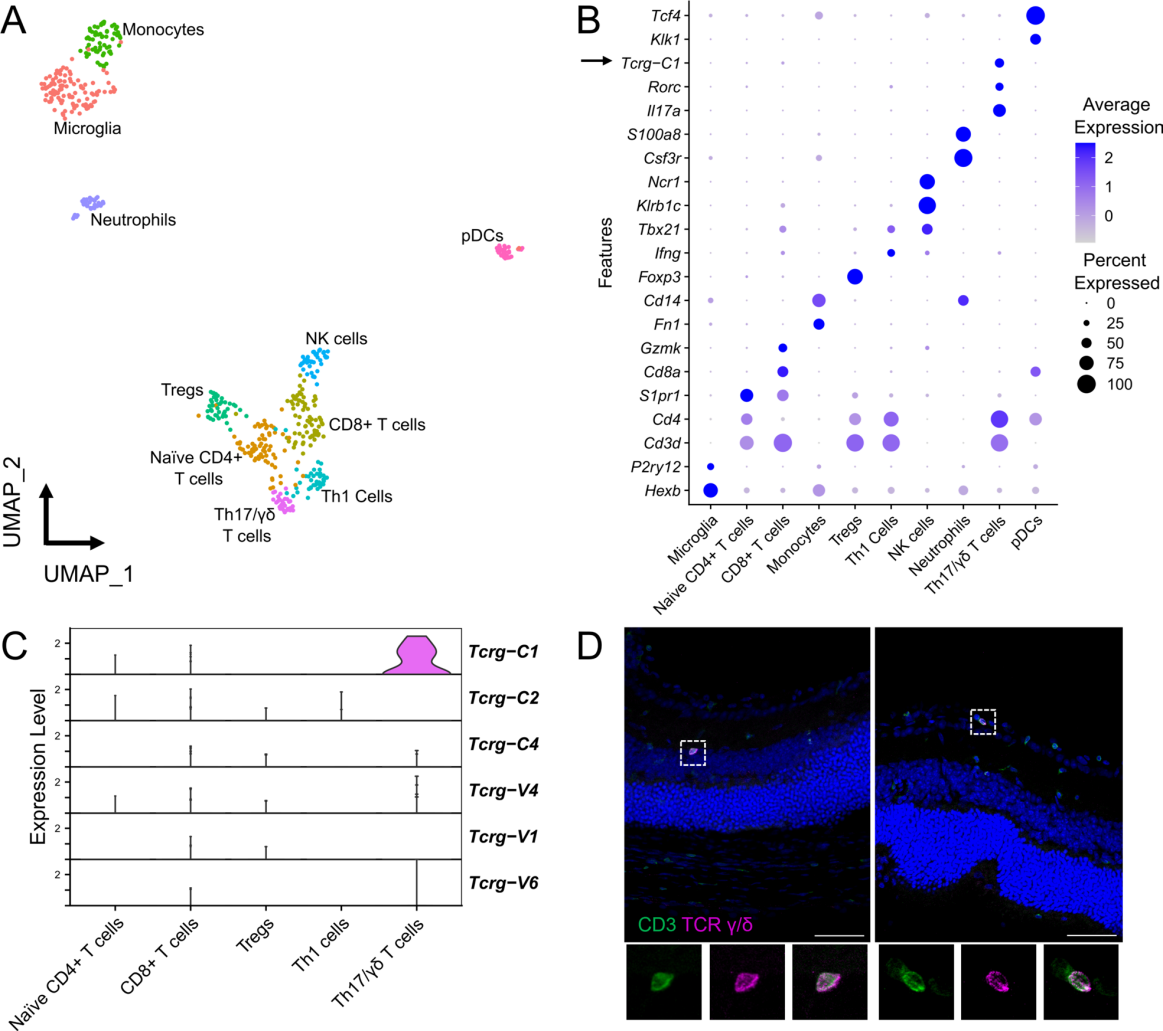
Single-cell analysis of infiltrating immune cells in inflamed retinas. (**A**) UMAP of the 548 immune cells captured in the two retinas with autoimmune uveitis. (**B**) Dot plot of selected immune cell marker genes. Black arrow highlights *Tcrg-C1* as a marker in Th17/γδ T cells. (**C**) Stacked violin plots showing expression of all TCR γ genes detected in the dataset. Greater expression of *Tcrg-C1* was present in the Th17/γδ cluster, suggesting γδ T cells are significant IL-17 producing cells in EAU retinas. (**D**) IHC of EAU retinas confirming presence of intraretinal CD3^+^ TCR γ/δ^+^ T cells (inset). Scale bar = 50 μm.

Focused data analysis on the retinal immune cell population further separated this into 10 clusters (Fig. 2A, Supplementary Table S3). An additional cell cluster of 66 cells containing presumed low quality cells and possible DCs was removed for downstream analysis. Unbiased marker gene identification showed the presence of microglia (*P2ry12*, *Tmem119; n* = *115 cells from all retinas*), monocytes (*Cd14*, *Fn1; n* = *65 cells*), neutrophils (*Csf3r*, *S100a8; n* = *34*), plasmacytoid dendritic cells (*Klk1*, *Tcf4; n* = *28 cells*), Th1 cells (*Cd3d*, *Cd4, Ifng; n* = *42 cells*), *Th17/γδ* cells (*Cd4*, *Il17a, Rorc, Tcrg-C1; n* = *29 cells*), CD8^+^ T cells (*Cd8a*, *Gzmb; n* = *70 cells*), Treg cells (*Foxp3*, *Il2ra; n* = *46 cells*), naïve CD4 + T cells (*Cd3d*, *Cd4*, *S1pr1; n* = *73 cells*) and natural killer (NK) cells (*Klrb1c*, *Ncr1; n* = *41 cells*) (Fig. 2B).

Interestingly, from unbiased marker gene identification *Tcrg-C1* was identified as a marker gene for the Th17 cell cluster (Fig. 2C), and immunostaining confirmed the presence of intraretinal γδ T cells (Fig. 2D), suggesting that γδ T cells are present and contribute to IL-17 production in the retina during uveitis. In addition, the T cell receptor (TCR) β gene *Trbv3* was defined as a significant marker gene for the Th1 cell cluster, and was also expressed in the Th17 cluster (Supplementary Fig. S2). However, it should be noted that the 10X 5’ kit is not able to distinguish between nascent and recombined TCR chains without specific TCR-seq.

### Differential gene expression analysis of retinal cells

**Figure 3:**
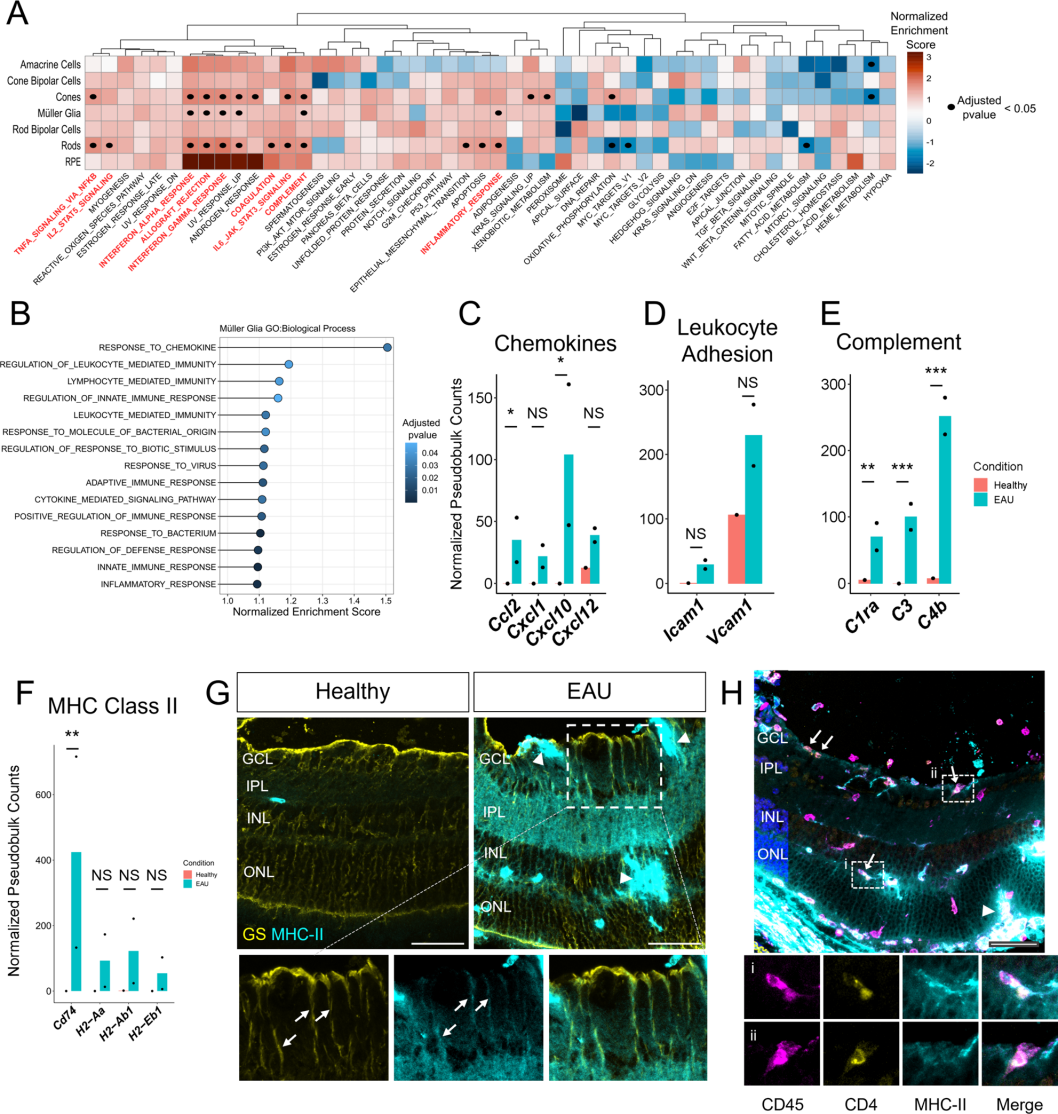
Activation of inflammation-associated gene sets in retinal cells during experimental autoimmune uveitis. (**A**) Summary heatmap of Gene Set Enrichment Analysis (GSEA) using the Molecular Signatures Database (MSigDB) Hallmark Gene Sets. The Müller glia, rods and cones, in particular, demonstrate significant upregulation of a range of proinflammatory gene sets, as denoted by black dots (adjusted p value < 0.05). Inflammation-associated gene sets that were significantly upregulated in at least one retinal cell type are highlighted in red. (**B**) Gene Ontology (GO) Biological Process gene sets that were significantly enriched in Müller glia. Leading edge analysis of significantly enriched gene sets shows upregulation of (**C**) chemokines, (**D**) complement factors/receptors, (**E**) leukocyte adhesion molecules and (**F**) MHC Class II genes. (ns) Not significant, * p < 0.05, ** p < 0.01, *** p < 0.001, DESeq2 Wald test. (**G**) Validation of MHC Class II protein expression by Müller glia in inflamed retina of EAU mice. Glutamine synthetase (GS) staining of Müller glia in yellow, MHC-II in cyan, scale bar = 50 μm. Inset images highlight an area showing co-staining of GS and MHC-II along Müller glia processes (arrows). Bright patches of confluent MHC-II^high^ only staining (arrow heads) likely represent infiltrating blood-derived professional antigen presenting cells (APCs). (**H**) Immunostaining of EAU retina for CD45, CD4 and MHC-II. Four arrows indicate areas where CD45^+^ CD4^+^ T cells colocalize with CD45^-^ MHC-II^+^ regions (corresponding to Müller glia processes as seen in panel (**G**)). These colocalizations appear distinct from the interaction between CD45^+^ CD4^+^ T cells and the MHC-II^high^ only blood-derived professional APCs (single arrowhead). Insets (i) and (ii): colocalization between CD45^+^ CD4^+^ T cells and CD45^-^ MHC-II^+^ processes in the outer plexiform layer and ganglion cell layer, respectively, suggestive of antigen presentation by the Müller glia.

To examine the immunological roles played by retinal cells during intraocular inflammation, we identified differentially expressed genes between the EAU and healthy retinas by pseudobulk differential expression analysis with DESeq2. All cell types displayed a number of differentially expressed genes apart from RPE, which was likely due to the small number of cells captured, as the number of differentially expressed genes appeared to correlate with the number of cells in the cluster (Supplementary Fig. S3; Supplementary Dataset 1). Preranked Gene Set Enrichment Analysis (GSEA) was then performed on differentially expressed genes with the Molecular Signatures Database (MSigDB) Hallmark gene sets. We found a general enrichment for inflammation-associated gene sets among all annotated retinal cell types, with rods, cones and Müller glia showing the greatest number of significantly enriched gene sets (Fig. 3A; Supplementary Dataset 2). Interferon-gamma (IFN-γ) and interferon-alpha (IFN-α) response gene sets were significantly upregulated by rods, cones and Müller glia, as well as showing positive enrichment in other retinal cell types, suggesting signalling through these cytokines is one of the major mechanisms by which retinal cells respond to inflammatory conditions during autoimmune uveitis directed against IRBP. Leading edge genes for inflammatory response gene sets in rods and cones were largely involved in upregulation of MHC Class I proteins (*H2-K1, H2-D1, B2m, Tap1*; Supplementary Fig. S4A) and the interferon regulatory factors (*Irf1, Irf8, Irf9*; Supplementary Fig. S4B). Interestingly, the rods and cones also demonstrate significant alterations in the expression of oxidative phosphorylation gene set, which was downregulated in rods but upregulated in cones. Upregulated oxidative phosphorylation leading edge genes in cones included cytochrome c oxidase subunits (*Cox4i1*, *Cox5b*, *Cox6b1*) and ATP synthase subunits (*Atp5h*, *Atp5g1*, *Atp5e*; Supplementary Fig. S4C). Downregulated oxidative phosphorylation leading edge genes in rods were primarily from the Mitochondrial Complex I (*Ndufa1*, *Ndufs2*, *Ndufa4;* Supplementary Fig. S4D). This observation provides a potential mechanistic link between retinal inflammation and impaired visual function via changes in photoreceptor ATP production.

To focus on gene expression changes in the Müller glia, which are thought to play a critical role in the regulation of inner blood-retinal barrier function, we performed additional preranked GSEA with the Gene Ontology (GO) Biological Process gene sets (Fig. 3B; Supplementary Dataset 3). This confirmed a predominant inflammatory response gene expression profile, with significantly enriched gene sets exclusively consisting of those associated with immunity. Leading edge analysis of the significantly upregulated GO Biological Process gene sets identified several chemokines (*Ccl2*, *Cxcl10*, *Cxcl1, Cxcl12*) and leukocyte adhesion molecules (*Icam1*, *Vcam1*) as contributing to enrichment scores (Fig. 3C,D). This suggests that Müller glia contribute to the recruitment and subsequent adhesion of circulating immune cells during autoimmune uveitis. Additionally, upregulation of complement factors and receptors (*C1ra*, *C3*, *C4b*) and MHC Class II genes (*Cd74*, *H2-Aa*, *H2-Ab1*, *H2-Eb1*) indicate their active roles in tissue damage and antigen presentation, respectively (Fig. 3E,F). The latter was validated at the protein level by immunohistochemical co-staining of the Müller glia marker, glutamine synthetase (GS) and MHC-II in EAU but not healthy retina (Fig. 3G). Additionally, non-leukocyte-mediated MHC-II expression likely attributed to Müller glia colocalized with CD45^+^CD4^+^ cells, indicating possible antigen presentation (Fig. 3H). However, this was relatively rare in comparison to leukocyte-mediated strong expression of MHC-II colocalizing with CD45^+^CD4^+^ cells, the major antigen presenting cells during experimental autoimmune uveitis.

### Predicted retinal cell-leukocyte interactions during EAU

**Figure 4:**
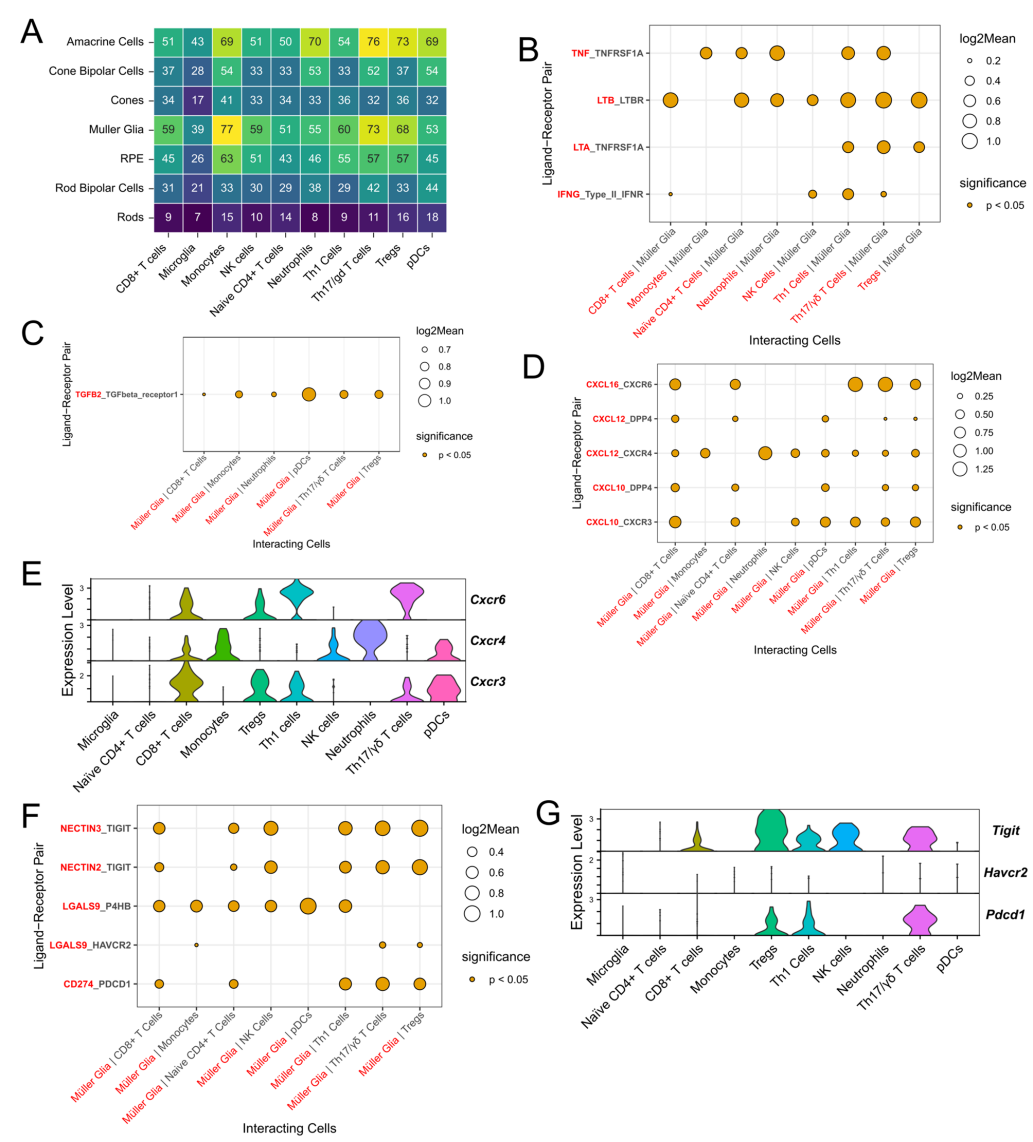
Immune ligand-receptor interactions between retinal cells and infiltrating immune cells. (**A**) Summary heatmap of ligand-receptor interactions between retinal cells and immune cells during experimental autoimmune uveitis using CellPhoneDB. (**B**) Extensive cytokine signalling between infiltrating lymphocytes and Müller glia was detected. Presence of a dot represents a significant interaction between two cell types, while the size of the dot is proportional to the mean expression level of the ligand-receptor pair. Ligands and ligand-expressing lymphocyte labels are coloured red, while receptors and receptor-expressing Müller glia are in black. IFN-γ produced by CD4 + T cells and NK cells is predicted to interact with the IFN-γ receptor expressed on Müller glia. CD4 + T cells also produce TNF-α and LT-α which may interact with their cognate receptors on Müller glia. (**C**) TGFB2 expressed by Muller glia was predicted to interact with TGFB receptors on CD8 + T cells, monocytes, neutrophils, pDCs, Th17/γδ T cells and Tregs. (**D**) Predicted chemokine-receptor interactions between Müller glia and lymphocytes. CXCL10, CXCL12 and CXCL16 expressed by Müller glia are predicted to interact with their receptors expressed by various immune cells. (**E**) Violin plots depicting expression of *Cxcr6*, *Cxcr4* and *Cxcr3* on immune cell subsets. *Cxcr6* was most highly expressed on Th1 and Th17/γδ T cells. Although *Cxcr4* was expressed on several immune cell subsets, highest expression appeared to be on monocytes and neutrophils. *Cxcr3* was most highly expressed on CD8 + T cells, Tregs, Th1 cells and pDCs. (**F**) Immune checkpoint ligand-receptor interactions between Müller glia and lymphocytes. Galectin-9 (*Lgals9*) expressed by Müller glia is predicted to interact with a several range of receptors on immune cells, including TIM-3 (*Havcr2*) expressed on Tregs. PD-L1 (*Cd274*) expression is upregulated in Müller glia during EAU and predicted to interact with its receptor PD-1 expressed on T cells. Nectin-2 and Nectin-3 expressed by Müller glia are also predicted to interact with their receptor TIGIT expressed on T cells and NK cells. (**G**) Violin plots showing expression of the checkpoint receptors *Tigit*, *Havcr2* and *Pdcd1*. *Tigit* was most strongly expressed by T and NK cell subsets, while *Pdcd1* showed higher expression on Tregs, Th1 and Th17/γδ T cells. *Havcr2* expression by comparison was lower across immune cell subsets.

Given that Müller glia are important regulators of the inner BRB and appear to increase expression of molecules associated with leukocyte recruitment and adhesion during EAU, we interrogated their potential ligand-receptor interactions with leukocytes using CellPhoneDB. Ligand-receptor interaction analysis across all annotated cell types during EAU suggested Müller glia were one of the main cell types interacting with immune cells in the retina (Fig. 4A). In total, 594 interactions were predicted between Müller glia and leukocytes. Of interest, IFN-γ, lymphotoxin-α, lymphotoxin-β and TNF-α produced by lymphocytes were predicted to interact with their corresponding receptors on Müller glia (Fig. 4B), further supporting the notion that these cytokines may drive the proinflammatory transcriptional changes. Müller glia also express TGF-β2, which is predicted to interact with its receptor primarily found on CD8^+^ and regulatory T cells (Fig. 4C, Supplementary Fig. S5).

Müller glial expression of the chemokines CXCL10, CXCL12 and CXCL16 was predicted to interact with leukocytes via their receptors CXCR3, CXCR4 and CXCR6, respectively (Fig. 4D,E). *Cxcl10* was also found to be significantly upregulated by Müller glia by differential expression between EAU and healthy retinas, thus indicating an acquired interaction that only occurs during autoimmune uveitis. The CXCL12-CXCR4 interaction was predicted to occur between Müller glia and all leukocyte subsets apart from naïve CD4 + T cells, with highest expression of *Cxcr4* occurring in neutrophils and monocytes (Fig. 4E). This may reflect a broad leukocyte recruitment strategy used by Müller glia during inner blood-retinal barrier breakdown. Finally, the CXCL16-CXCR6 interaction was predicted to occur exclusively between Müller glia and T cells, with the highest expression of *Cxcr6* occurring in the Th1 and Th17/γδ subsets (Fig. 4E). Thus, this may be an important signalling axis for recruiting pathogenic T cells to the retina.

Interestingly, Müller glia appear to provide a complex range of costimulatory and coinhibitory signals to the infiltrating T cells and natural killer (NK) cells in the retina via a number of different immune checkpoint ligand-receptor interactions (Fig. 4F,G). For instance, the inhibitory galectin-9-TIM-3 interaction was to occur between Müller glia and Th17 cells, Tregs, microglia and monocytes. Furthermore, inhibitory interactions between Müller glia and all T cell subsets were predicted to occur through the PD-L1-PD-1, nectin-2-TIGIT and nectin-3-TIGIT axes. In contrast, Müller glia-derived galectin-9 was also predicted to interact with the cell surface protein disulfide-isomerase, P4HB, on Th1, CD8 + T cells, naïve T cells, Tregs, NK cells, monocytes, microglia and pDCs. This interaction may enhance T cell migration in the retina^14^; however, the role of this interaction in myeloid cells is unclear. Together, these results suggest that Muller glia may directly provide immunomodulatory signals to infiltrating immune cells during uveitis.

### Transcriptomic analysis of the retinal pigment epithelium during EAU

**Figure 5:**
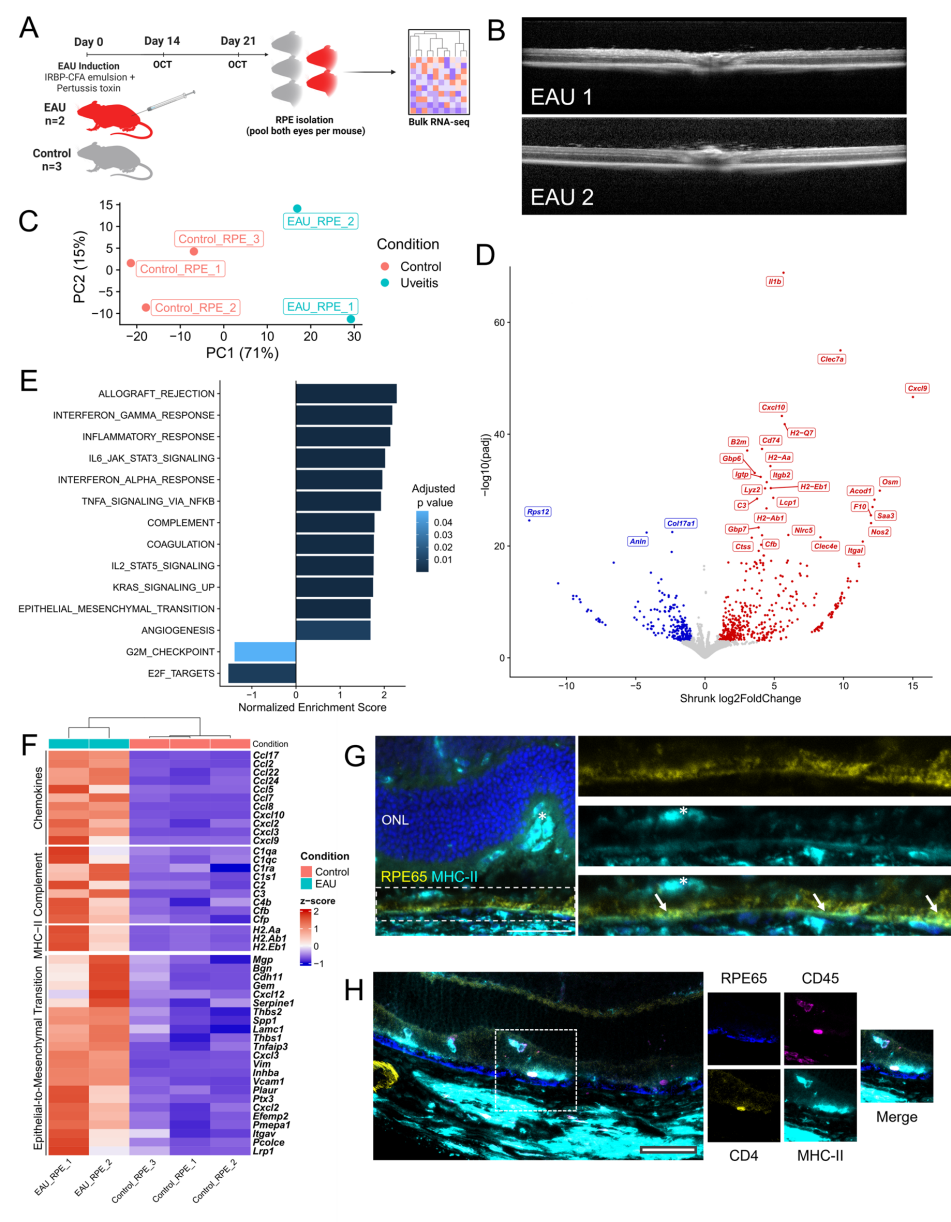
Bulk RNA-seq and immunohistochemical analysis of the retinal pigment epithelium (RPE) during homeostasis and experimental autoimmune uveitis. (**A**) Experimental outline of bulk RNA-seq of EAU RPE, with cells harvested from eyecups at 21 days post-immunisation. (**B**) OCT of eyes from each of two mice that provided the EAU RPE samples showing characteristic features of posterior uveitis. (**C**) PCA plot of analysed samples showing variation between samples is primarily by condition. (**D**) Volcano plot showing the 824 significantly differentially expressed genes from DESeq2 analysis. Top 30 differentially expressed genes are labelled. (**E**) Preranked GSEA results of EAU RPE showing significantly enriched gene sets from the Molecular Signatures Database Hallmark gene sets. (**F**) Heatmap of leading edge genes from inflammation-associated gene sets. All displayed genes are significantly differentially expressed between EAU and healthy RPE (p < 0.05, DESeq2 Wald Test). Inflammation-associated leading edge genes showed upregulation of chemokines, complement factors and MHC-II genes. Leading edge analysis of the Epithelial-to-Mesenchymal Transition (EMT) signature genes included extracellular matrix proteins and matrix metalloproteinases (*Mgp*, *Bgn*, *Pcolce*, *Serpine1*), as well as typical transition markers (*Vim*, *Inhba*) and adhesion molecules (*Vcam1*, *Itgav*). (**G**) Immunohistochemistry co-staining of RPE65 (yellow) and MHC-II (cyan) in EAU retina. Left, Hoechst staining of photoreceptor nuclei (blue); asterisk (*) highlights a cluster of infiltrating subretinal MHC-II^hi^ immune cells. Dashed box denotes the RPE layer. Right top, RPE65 staining of RPE cells. Right middle, MHC-II staining predominantly at the basal surface of the RPE layer; asterisk indicating MHC-II^hi^ immune cell located on apical side of RPE. Right bottom, merge of RPE65 and MHC-II staining with arrows indicating regions of strongest co-staining, suggestive of expression of MHC-II by the RPE during retinal inflammation. (**H**) Inset: an infiltrating (subretinal) CD45 + CD4 + T cell on the apical surface of the RPE layer. However, we did not identify overlap between MHC-II and RPE65 staining, suggesting this was not RPE-mediated. No evidence of colocalization of CD45^+^ CD4^+^ cells with RPE at the basal surface was found in analyzed sections. Scale bars = 50 μm.

The healthy RPE monolayer with tight junctions consisting of claudins, occludin and other junctional adhesion molecules (JAMs) between hexagonal cells constitute the physical outer blood-retinal barrier. Due to the technical limitation of capturing only a small number of RPE cells during retinal harvesting for single cell RNA-sequencing, we performed separate bulk RNA-seq analysis of pooled RPE isolated from control (n = 3) and EAU (n = 2) posterior eyecups (Fig. 5A,B). Principal Component Analysis (PCA) revealed that the majority of variation between samples was driven by the condition, with EAU and healthy RPE showing good segregation (Fig. 5C).

Using a similar differential expression pipeline with DESeq2 and subsequent pre-ranked GSEA, we found 824 differentially expressed genes between EAU and healthy RPE (Fig. 5D; Supplementary Dataset 4) and a similar enrichment of pro-inflammatory gene sets (Fig. 5E; Supplementary Dataset 5). Leading edge analysis of the pro-inflammatory gene sets again revealed significant upregulation of a number of chemokines, complement and MHC-II genes (Fig. 5F). As molecular evidence of breakdown of the outer blood-retinal barrier, we also found significant enrichment of the ‘Epithelial-to-Mesenchymal Transition’ (EMT) gene set in the RPE of eyes with active uveitis. Leading edge genes included mesenchymal markers (*Vim*, *Vcam1*, *Spp1*, *Inhba*), as well as proteases and extracellular matrix components (*Mgp*, *Bgn*, *Serpine1*, *Pcolce*) (Fig. 5F).

As with Müller glia, MHC Class II expression by RPE was validated at the protein level by IHC, with expression colocalizing with RPE65 predominantly on the basal side of the RPE layer during EAU (Fig. 5G). We were able to identify CD45^+^ CD4^+^ T cells among infiltrating immune cells in the subretinal space (Fig. 5H). However, unlike for Müller glia, we were unable to identify colocalization between such CD4^+^ T cells and MHC-II^+^ RPE cells within the analysed sections, which would suggest this to be a relatively rare interaction.

## Discussion

Here, we present the first single cell transcriptomic characterization of immunization-induced experimental autoimmune uveitis in mice as a model for human posterior uveitis. Retinas with active grade 2 EAU at 21 days post-immunization were compared against healthy control by scRNA-seq profiling of retinal cell populations and immune infiltrate, revealing changes in a range of immune-related genes associated with breakdown of the blood-retinal barrier. Several manifestations of human posterior uveitis have been shown to be driven by a mixed Th1/Th17 immune response through cytokine and flow cytometric analyses^15–20^. The mouse EAU model of retinal inflammation has been shown to be Th1/Th17-driven^21,22^, thus recapitulating the inflammatory cytokine milieu in human disease and is suitable for exploring clinically relevant immune interactions.

Our results reveal a major role for Müller glia in regulating inner BRB function through their production of chemokines and proinflammatory cytokines, as well as immune checkpoint ligand-receptor interactions with leukocytes during retinal inflammation. In addition, we supplemented the analysis with bulk RNA-seq of RPE from EAU to define gene expression changes in the main cellular constituent of the outer BRB. This identified a similar shift of the RPE toward a leukocyte-recruiting and antigen-presenting state during retinal inflammation. Using GSEA, we showed that inflammation-associated gene sets are upregulated in Müller glia and other retinal cells during EAU. Similar pathways were also found to be upregulated in the RPE. Notably, the ‘Response to Interferon Gamma’ gene set was significantly upregulated in both Müller glia and RPE, in keeping with previous observations in the *Aire* knockout mouse retina, which displayed a purely Th1-driven uveoretinitis caused by abolished central tolerance to retinal antigens^23^. Additionally, recent work has revealed an IFN-γ-responsive population of Müller glia in the retina which is poised to respond to injury in an optic nerve crush model^24^. Ligand-receptor interaction analysis showed that infiltrating T cells producing IFN-γ, TNF-α and lymphotoxins-α and -β are likely to be the main source of these proinflammatory cytokine signals to the Müller glia.

Interestingly, we also found that Müller glia moderately upregulate *Cxcl1*, a chemokine that predominantly recruits neutrophils via CXCR1/CXCR2 receptors and is a target for upregulation by IL-17A. Indeed, a population of infiltrating neutrophils was present in the EAU retinas, whereas previously reported *Aire*^-/-^ retinas did not appear to have a notable neutrophil infiltrate^23^. This suggests that Müller glia may alter gene expression broadly in response to IFN-γ, while IL-17A may subtly shape the chemokine expression profile during uveitis. This hypothesis is corroborated by previous work in a mouse model of central nervous system (CNS) neuroinflammation describing gliosis without blood–brain barrier breakdown, leukocyte infiltration or tissue damage in a mouse overexpressing IL-17A in astrocytes under the control of a GFAP promoter^25^.

Ligand-receptor interaction analysis indicates that Müller glia-produced chemokines, CXCL10, CXCL12 and CXCL16, are likely to be important in the recruitment of immune cells to the retina during inflammation. CXCL10 (also known as IP-10) is a chemokine that is upregulated in response to IFN-γ to target a variety of immune cells. Our data suggest the main cells targeted by CXCL10 are likely to be all T cell subsets, as well as NK cells and plasmacytoid dendritic cells (pDCs). CXCL10 levels have also been found to be increased in ocular fluids from patients with uveitis, suggesting these interactions parallel those in human disease^26,27^. CXCL12 expressed by Müller glia has an even broader range of target cells, with all infiltrating immune cells apart from naïve T cells being implicated. Its target receptor, CXCR4, has previously been shown to be important in leukocyte trafficking in both an ovalbumin-induced uveitis model and an adoptive transfer EAU model^28,29^. In both cases, the CXCR4 antagonist, AMD3100, attenuated leukocyte infiltration into the eye during disease induction, possibly due to the near pan-leukocyte targeting activity as seen in our data. Finally, the CXCL16-CXCR6 interaction was predicted to occur exclusively between Müller glia and T cells. CXCR6 has previously been reported as a marker for pathogenic effector T cell populations in experimental autoimmune encephalomyelitis (EAE)^30^. These effector T cell populations were preferentially recruited to the CNS where CXCL16 expression was found to be greater, thus a similar mechanism of T cell recruitment may exist in the retina.

Our results provide evidence that both Müller glia and RPE may be capable of antigen presentation via MHC Class II during retinal inflammation. Some controversy surrounds non-leukocyte MHC-II expression in the retina, as a previous study concluded that MHC-II expression was not attributable to retinal glial cells or RPE based on immunohistochemical co-staining for select markers^31^. However, our findings are consistent with previous in vitro studies of RPE cells and single cell transcriptomic analysis of *Aire*^−/−^ retina suggesting MHC-II expression during inflammation^23,32–34^. Retinal glia^35^ and RPE^33,36^ have been shown to be able to stimulate T cells in culture following treatment with inflammatory cytokines, but in vivo evidence is scarce. MHC-II-mediated interactions between Müller glia/RPE and T cells may be rare, spatially restricted, transient or occur at specific phases of the disease course that make it challenging to find evidence of by IHC. We identified putative spatial colocalization of CD45^-^ MHC-II^+^ Müller glia with CD4^+^ T cells in EAU retina, but functional antigen presentation is difficult to prove. Furthermore, as we and others have shown, MHC-II is expressed strongly and widely by infiltrating leukocytes, likely making these professional antigen presenting cells more important for T cell stimulation during retinal autoimmunity^37^. Thus, the functional consequences of in vivo MHC-II expression by Muller glia and RPE require further investigation. Even so, these findings may have significant implications for retinal gene therapy using adeno-associated viral (AAV) vectors since AAV transduction of RPE and Müller glia could lead to viral antigen presentation and clinically undesirable retinal immune cell infiltration^38^. Whether the retinal microglia are also capable of acting as antigen presenting cells remains uncertain, as it is currently difficult to distinguish infiltrating monocyte-derived macrophages from activated microglia without strict lineage tracing^39,40^.

In addition to this possible antigen-presenting role, our data also revealed that Müller glia may provide co-inhibitory signals to infiltrating lymphocytes through expression of the immune checkpoint ligands, PD-L1 (*Cd274*), galectin-9 (*Lgals9*), nectin-2 and nectin-3. PD-L1 has been studied as one of the mechanisms by which pigmented cells of the uvea are capable of suppressing T cell activation in vitro, and is upregulated in response to inflammatory cytokines^6,7,41^. Our observation of Müller glia-lymphocyte crosstalk via PD-L1 to PD-1 interaction provides a potential mechanistic explanation for the observation of spontaneous BRB breakdown and uveitis in patients receiving systemic PD-1 checkpoint inhibitors for cancer^42^. Galectin-9 is a member of a class of β-galactoside-binding proteins that often have pleiotropic effects with multiple binding partners^43^. For instance, the interaction of galectin-9 with TIM-3 functions as an inhibitory immune checkpoint with some beneficial effects in mouse models of autoimmune disease (including in the CNS), but in some cases the effect was not TIM-3-dependent, which highlights the complexity of galectin-9 signalling^44,45^. The role of galectin-9 in retinal inflammation remains poorly understood and potential benefit of targeted therapeutic intervention merits further investigation.

In order to capture an unbiased landscape of gene expression across retinal and immune cell populations, we analysed whole dissociated retinas. However, one of the limitations of this approach has been the capture of a relatively modest number of immune cells, an overrepresentation of rods, cones and bipolar cell, and underrepresentation of retinal endothelial cells, pericytes and perivascular macrophages which may coordinate their activities with Müller glia to regulate inner BRB function. Future work may focus on these rarer cell populations by enriching for non-neuronal populations prior to scRNA-seq. In addition, our data captures the transcriptional profile and cellular interactions during active retinal inflammation in EAU, while future work to characterize the temporal sequence of gene expression changes in BRB cells and infiltrating leukocytes as the disease onsets and subsides over the course of 6–8 weeks could provide further mechanistic insights.

Nonetheless, our data provide a wholistic molecular understanding of key local immune interactions during autoimmune retinal inflammation, which can help guide the development of targeted immunomodulatory interventions. While both Müller glia and RPE upregulate expression of chemokines that recruit immune cells to the retina and drive ocular inflammation, Müller glia also take part in numerous immune ligand-receptor interactions with the infiltrating leukocytes, which are likely to shape the severity and duration of tissue inflammation. Both Müller glia and RPE were found to upregulate MHC Class II, indicating a possible acquired antigen-presenting function during retinal inflammation. Furthermore, we observed an epithelial-to-mesenchymal transition signature in the inflamed RPE, which would contribute to outer BRB breakdown and correlate with clinical observations of pigment migration and secondary choroidal neovascularisation in posterior uveitis. Future work will aim to validate and characterize the functional importance of key BRB-leukocyte interactions at the protein level, with the aim of developing highly targeted therapeutics for uveitis and other neuroinflammatory diseases.

## Materials and methods

### Animals

All animals were maintained by the Biomedical Science Division, University of Oxford, UK. Mice were housed in a 12-h light–dark cycle, with food and water available ad libitum. All animal procedures were approved by the Oxford University Animal Welfare and Ethical Review Board (AWERB) and the UK Home Office. All work was undertaken in accordance with the Association for Research in Vision and Ophthalmology (ARVO) guidelines for the humane use of laboratory animals in ophthalmic research. Results are reported in accordance with the ARRIVE guidelines. Immunisations were performed under inhalational anaesthesia with isoflurane, and imaging was performed under general anaesthesia by intraperitoneal injection of a mixture of 80 mg/kg ketamine and 10 mg/kg xylazine.

### Induction, imaging and scoring of EAU

Induction of EAU was performed by immunising 6 to 10-week-old, female C57BL/6 J mice with 500 μg of IRBP1-20 peptide (GPTHLFQPSLVLDMAKVLLD; synthesised by Merck) emulsified in a 1:1 ratio with Complete Freund’s Adjuvant (CFA) containing 2.5 mg/ml heat-killed *Mycobacterium tuberculosis* (MTb). Mice received 100 μl of emulsion as two 50 μl injections, one in each hind flank. Mice were then injected with 1.5 μg Bordetella pertussis toxin as a 100 μl intraperitoneal injection. Littermate control mice were maintained untreated. At specified timepoints, mice retinas were imaged in vivo using scanning laser ophthalmoscopy (SLO) and optical coherence tomography (OCT). The level of retinal inflammation was then graded based on a previously published protocol^13^ by two independent scorers who were blinded to the treatment of the mouse.

### Immunohistochemistry and confocal microscopy

Mice were euthanised and eyes enucleated and cleaned of excess tissue. Eyes were placed in 4% paraformaldehyde on ice for 20 min, followed by a brief rinse in PBS and then cryoprotected in a sucrose gradient (10%, 20%, 30%) at 4 °C. Eyes were then briefly incubated in optimal cutting temperature medium and frozen in moulds on dry ice. 16 μm sections were cut at − 20 °C with a cryotome and positioned onto Superfrost plus slides (VWR). Slides were washed in PBS, then blocked with 5% BSA, 5% serum of the secondary antibody host. Slides were then incubated with primary antibodies (full list of antibodies provided in Supplementary Table S4). overnight at 4 °C in a solution containing 1% BSA, 1% serum in PBS. Slides were washed with 0.05% Tween-20 in PBS, rinsed in PBS then, when necessary, stained with secondary antibodies under dark conditions for 2 h at room temperature. Slides were then briefly washed with 0.05% Tween-20 in PBS before counterstaining with Hoechst for 30 min in the dark. Coverslips were mounted with ProLong Diamond and sealed. Z-stack images of sections were captured on a LSM 710 confocal microscope (Zeiss) and data analysed in ImageJ.

### Preparation of retinas for single-cell RNA-sequencing

After imaging at day 21 post-immunisation, mice were euthanised by cervical dislocation and eyes were enucleated. The retinas were carefully dissected out and placed into Hanks’ Balanced Salt Solution (HBSS) containing 10 mM HEPES on ice for transport. After all retinas were collected, they were dissociated using the Worthington Papain Dissociation system. Retinas were placed in a solution of 20 U/ml papain, 0.005% DNase I with 1 mM L-cysteine and 5 mM EDTA in Earle’s Balanced Salt Solution (EBSS), for 10 min at 37 °C with frequent, gentle agitation. Samples were then diluted by addition of 500 μl of EBSS to inactivate the papain and centrifuged at 300 × *g* for 5 min at room temperature. Pellets were resuspended in 525 μl of a solution containing 1 mg/ml ovomucoid and BSA and 100 U/ml DNase I in EBSS. The resulting suspension was carefully layered over 500 μl ovomucoid/BSA solution and centrifuged at 70 × *g* for 6 min. Supernatant was discarded and cells were resuspended in a solution of PBS containing 0.04% BSA.

### Single-cell RNA-seq library generation and processing

Library generation from dissociated retinas and initial data processing was performed by the Oxford Genomics Centre. scRNAseq transcriptome processing was performed using the Chromium 10X system involving GEM generation, post GEM-generation clean-up, cDNA amplification and DNA quantification. Chromium Single Cell Reagent Kits solution (10X SC RNA CITE-TSC, 10X SC RNA 5pr v2.0 Chemistry) was used to deliver a scalable microfluidic platform for digital scRNA-seq by profiling 500–10,000 individual cells per sample. A pool of ~ 3,500,000 10 × Barcodes were sampled separately to index each cell’s transcriptome. Libraries were generated and sequenced from the cDNAs and 10 × Barcodes were used to associate individual reads back to the individual partitions. The library was sequenced using the Illumina NovaSeq platform.

For initial data processing, Illumina’s bcl2fastq and cellranger mkfastq demultiplexes were used to convert the raw base call (BCL) files generated by Illumina sequencers into FASTQ files. Cellranger was used to perform alignment, filtering, barcode counting, and UMI counting from FASTQ files. This generated feature-barcode matrices for each sample, which was used for downstream analyses.

### Analysis of single-cell RNA-seq data

Analysis was performed with the R programming language. The SoupX package^46^ was used to correct raw feature-barcode matrices for ambient RNA contamination. Corrected matrices were then analysed using the Seurat package^47^. Commonly used QC metrics, such as UMI count, number of features, percentage mitochondrial RNA and percentage ribosomal RNA were used to filter out low quality cells. Samples were then normalised using the SCTransform function in Seurat, and integrated to remove batch effects and generate a single feature-barcode matrix.

The integrated matrix was then passed through standard dimensionality reduction and clustering pipelines in Seurat. Briefly, Principal Component Analysis (PCA) was used to determine dataset dimensionality, followed by shared nearest-neighbour graph construction and dimensionality reduction with the Uniform Manifold Approximation Projection (UMAP) method. Visualisations were performed using in-built functions in Seurat. Differential expression was performed by pseudobulking each identified cell type by sample and analysing with the DESeq2 package^48^. Ligand-receptor interaction analysis was performed with CellPhoneDB package in Python using the statistical analysis method^49^. DESeq2 and CellPhoneDB heatmaps were produced using the pheatmap package in R.

### RPE isolation

RPE were isolated according to a previously established protocol^50^. Briefly, mice were euthanized and eyes were enucleated and cleaned of excess tissue. The cornea and lens were dissected out and eyecups were placed in a solution of HBSS containing 10 mM HEPES and 1 mg/mL hyaluronidase for 45 min at 37, 5% CO_2_. Eyecups were then placed in a solution of 10 mM HEPES in HBSS (with Ca/Mg), on ice for 30 min. Retinas were removed and the remaining eyecup was incubated in 0.25% trypsin–EDTA solution at 37C, 5% CO_2_ for 45 min. RPE were isolated by gently shaking eyecups to dislodge RPE cells into a solution of 10 mM HEPES in HBSS (with Ca/Mg). Each sample contained RPE from both eyes from one mouse.

### Bulk RNA-seq of RPE

RNA was extracted from samples using the Qiagen microRNeasy kit according to manufacturer’s protocol. Sequencing libraries were prepared using the NEBNext^®^ Ultra™ II Directional RNA Library Prep Kit for Illumina® according to the manufacturer’s protocol. During this process, the libraries were indexed using NEBNext® Multiplex Oligos for Illumina^®^ (Index Primers Set 4). The prepared libraries were quantified via a fluorometric method involving an Invitrogen Qubit dsDNA assay and qualified using electrophoretic separation on the Agilent BioAnalyzer 2100. This concentration and sizing information was used to calculate the molarity of each sample prior to pooling and sequencing of 150 bp paired-end reads using the Illumina NovaSeq 6000 platform. Sequencing reads were processed using the Nextflow (v22.04.5)^51^ nf-core RNA-Seq pipline (v3.8)^52^. The quality of reads was assessed using FastQC where a mean Phred score > 30 was observed across the full length of reads across all samples. Reads were then pseudo aligned to the mouse reference genome (GRCm38) using Salmon (v1.5.2)^53^ with > 65% of reads aligning across all samples. Transcript counts were converted to gene level counts using the tx2gene function from the R package tximport^54^. These were used for subsequent differential expression analyses of RPE in healthy vs uveitis with DESeq2. Tx2gene was also used to generate length normalised (transcript per million, TPM) counts for subsequent plotting.

### Supplementary Information

Supplementary Figures.

Supplementary Information 1.

Supplementary Information 2.

Supplementary Information 3.

Supplementary Information 4.

Supplementary Information 5.

## Data Availability

Code used for analysis is available on GitHub (https://github.com/JoelQuinn). Raw and processed single cell RNA-seq and bulk RNA-seq files have been deposited in the NCBI’s Gene Expression Omnibus and are accessible through GEO Series accession number GSE241700 (https://www.ncbi.nlm.nih.gov/geo/query/acc.cgi?acc=GSE241700).
