# Supplementary material for: Single-cell transcriptomic analysis of retinal immune regulation and blood-retinal barrier function during experimental autoimmune uveitis
Source: Sci Rep. 2024 Aug 28;14:20033. doi: 10.1038/s41598-024-68401-y (PMC11358488; doi:10.1038/s41598-024-68401-y)
Supplement: Supplementary file 1 — Supplementary Figures. [file 41598_2024_68401_MOESM1_ESM.pdf]

Supplementary Information

Single-cell transcriptomic analysis of retinal immune regulation and blood-retinal barrier function during experimental autoimmune uveitis

Joel Quinn[1], Ahmed Salman[1], Christopher Paluch[2,3,4], Matthew Jackson-Wood[2], Michelle E McClements[1], Jian Luo[5], Simon J Davis[3,4], Richard J Cornall[6,7], Robert E MacLaren[1,8], Calliope A Dendrou[9,10], Kanmin Xue[1,8,*]

Kanmin Xue
Email: enquiries@eye.ox.ac.uk

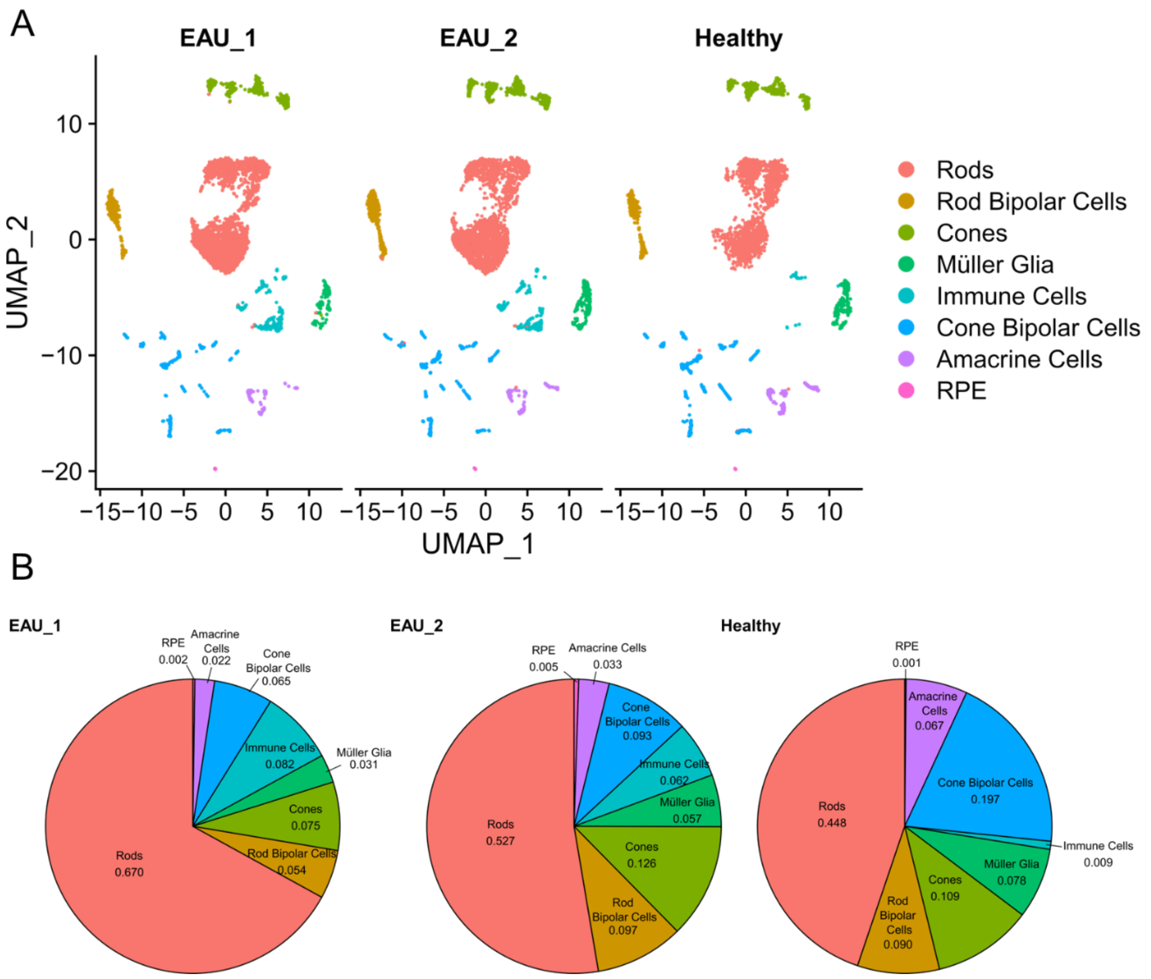

Fig. S1. Distribution of cell types by sample. (A) UMAPs split by sample showing presence of each annotated cell type in each sample. EAU samples contributed greatest to the immune cell cluster, as expected. (B) Proportions of each cell type by sample.

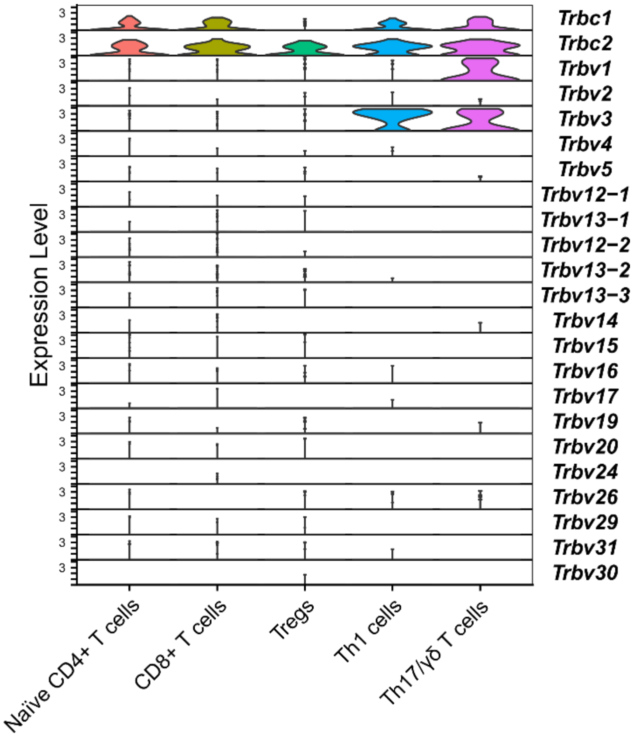

Fig. S2. TCR-β gene expression among T cell subsets. *Trbv3* was found to be a marker gene for the Th1 cell subset, and was also expressed by Th17/γδ T cells.

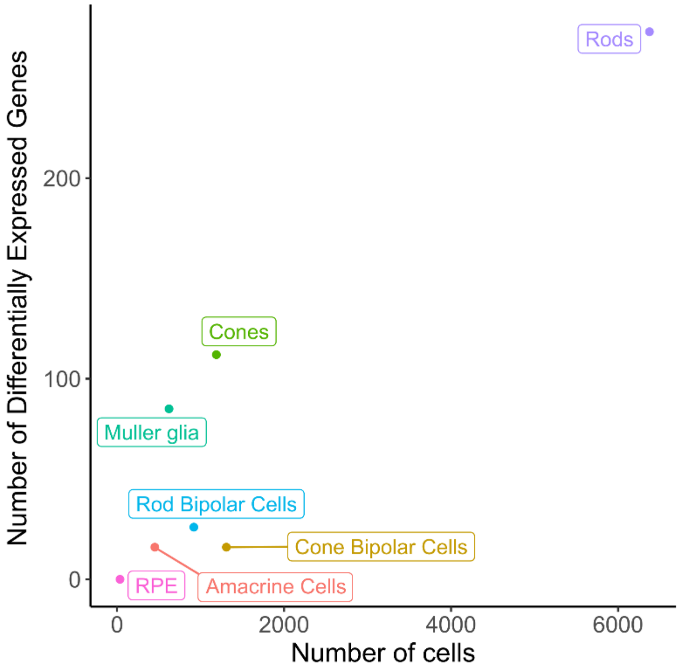

Fig. S3. Relationship between number of differentially expressed genes vs number of cells in cluster.

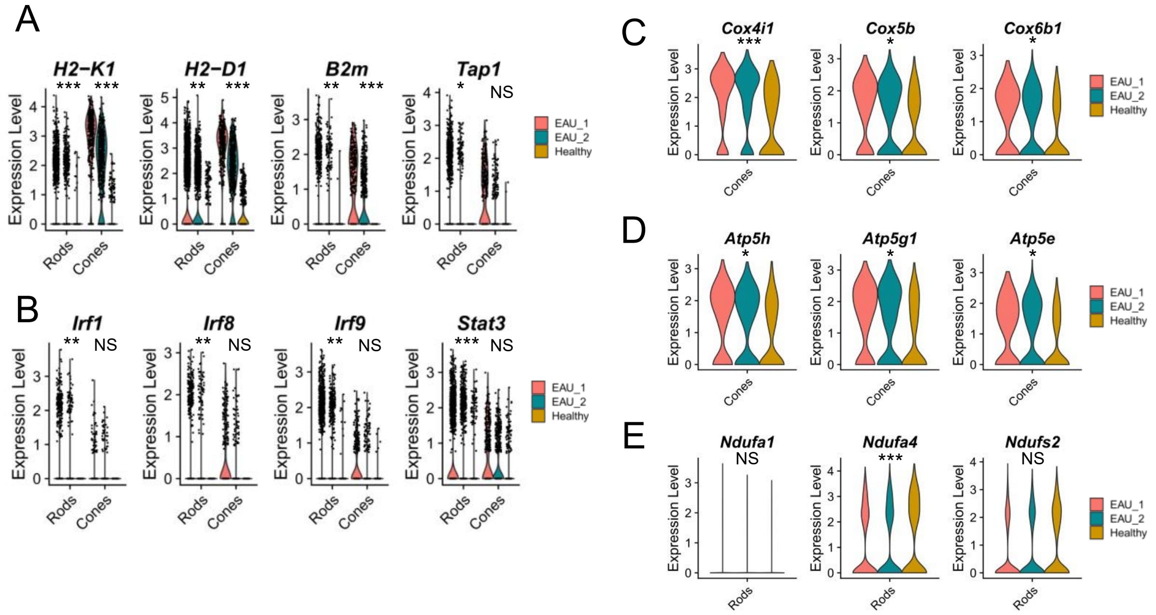

Fig. S4. Leading edge genes for significantly enriched gene sets by GSEA. Genes contributing to enrichment of inflammatory gene sets included (A) MHC-I antigen presentation pathway genes and (B) interferon regulatory factors. Enrichment of the Oxidative Phosphorylation gene set in cone photoreceptors was primarily due to upregulation of (C) cytochrome c oxidase genes and (D) ATP synthase genes during EAU. (E) Downregulation of Mitochondrial Complex I genes were the primary contributors to negative enrichment of the Oxidative Phosphorylation gene set in rod photoreceptors. * $p < 0.05$, ** $p < 0.01$, *** $p < 0.001$, DESeq2 Wald test.

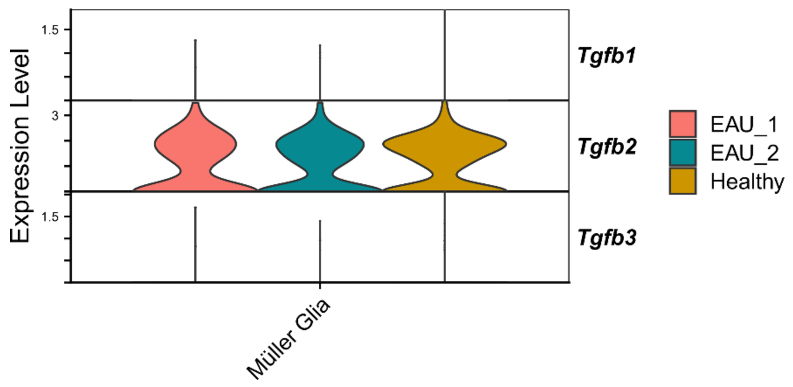

Fig. S5. TGF-b isoform expression by Müller glia for each sample.

Table S1. Summary of cell numbers for single-cell RNAseq by sample.

Sample ID	Cell number
EAU_1	4029
EAU_2	4108
Healthy	3379
**Total**	**11516**

Table S2. Summary of cell numbers by annotated cell type per sample.

Cell type	EAU_1	EAU_2	Healthy	Total
Rods	2699	2163	1514	**6376**
Rod Bipolar Cells	216	399	304	**919**
Cones	303	517	369	**1189**
Muller Glia	125	235	262	**622**
Immune Cells	329	255	30	**614**
Cone Bipolar Cells	261	381	667	**1309**
Amacrine Cells	87	137	228	**452**
RPE	9	21	5	**35**

Table S3. Summary of immune cell numbers by annotated cell type per sample.

Cell type	EAU_1	EAU_2	Healthy	Total
CD8+ T cells	46	24	0	**70**
Microglia	46	47	22	**115**
Monocytes	44	20	1	**65**
Naive CD4+ T cells	36	35	2	**73**
Neutrophils	27	6	1	**34**
NK cells	30	11	0	**41**
pDCs	12	16	0	**28**
Th1 Cells	22	20	0	**42**
Th17/gd T cells	9	20	0	**29**
Tregs	26	20	0	**46**

Table S4. Antibodies used in this study.

Antibody	Clone	Supplier Product	Dilution
Rat anti-mouse CD45	S18009D	BioLegend #160302	1:200
Rat anti-mouse I-A/I-E	M5/114.15.2	BioLegend #107601	1:100
Rabbit anti-mouse RPE65	EPR22579-44	abcam #ab231782	1:200
Rabbit anti-mouse glutamine synthetase	Polyclonal	abcam #ab228590	1:200
Rat anti-mouse CD45 AlexaFluor 594	S18009D	BioLegend #160307	1:100
Rat anti-mouse I-A/I-E AlexaFluor 647	M5/114.15.2	BioLegend #107617	1:100
Rat ant-mouse CD4 AlexaFluor 488	GK1.5	BioLegend #100425	1:100
Armenian hamster anti-mouse TCR g/d	GL3	BioLegend #118101	1:100
Rat anti-mouse CD3 AlexaFluor 488	17A2	BioLegend #100212	1:100
Goat anti-rat IgG AlexaFluor 647	Polyclonal	Invitrogen #A21247	1:400
Goat anti-rabbit AlexaFluor 568	Polyclonal	Invitrogen #A11036	1:400
Goat anti-armenian hamster AlexaFluor 568	Polyclonal	Invitrogen #A78965	1:400
Goat anti-rabbit IgG AlexaFluor 405	Polyclonal	Abcam #ab175652	1:400
